# Patterns of biophonic periodicity on coral reefs in the Great Barrier Reef

**DOI:** 10.1038/s41598-017-15838-z

**Published:** 2017-12-12

**Authors:** Jamie N. McWilliam, Robert D. McCauley, Christine Erbe, Miles J. G. Parsons

**Affiliations:** Centre for Marine Science and Technology, Curtin University, GPO Box U1987, Perth, Western Australia 6845 Australia

## Abstract

The coral reefs surrounding Lizard Island in the Great Barrier Reef have a diverse soundscape that contains an array of bioacoustic phenomena, notably choruses produced by fishes. Six fish choruses identified around Lizard Island exhibited distinctive spatial and temporal patterns from 2014 to 2016. Several choruses displayed site fidelity, indicating that particular sites may represent important habitat for fish species, such as fish spawning aggregations sites. The choruses displayed a broad range of periodicities, from diel to annual, which provides new insights into the ecology of vocalising reef fish species and the surrounding ecosystem. All choruses were affected by one or more environmental variables including temperature and moonlight, the latter of which had a significant influence on the timing and received sound levels. These findings highlight the utility of passive acoustic tools for long-term monitoring and management of coral reefs, which is highly relevant in light of recent global disturbance events, particularly coral bleaching.

## Introduction

Environmental rhythms strongly influence the presence, diversity and dispersal of organisms in ecosystems, where cyclical patterns are a fundamental part of natural systems^1^. In the marine environment, synchronising with environmental patterns in tides, temperature and season helps increase a species’ chance of survival^2^. One such adaptation and well-known phenomenon is the spawning aggregation of fishes, defined as ‘any temporary aggregation formed by fishes that have migrated for the specific purpose of spawning’^3^.

Globally, 164 species of reef fishes across 26 families have been identified as forming spawning aggregations^3–5^. The commencement of spawning in fish has been shown to be influenced by a combination of environmental cues, notably water temperature^6^, tidal regimes^7,8^ and lunar phase^9,10^. These may act synergistically or antagonistically to stimulate changes in behaviour^11^. Fish spawning behaviour is also strongly influenced by circadian and circalunar rhythms, where solar and lunar photoperiods are key triggers of gonadal activation and diurnal breeding in many tropical reef species^12,13^.

Several species that form spawning aggregations produce sound during mating, including Pomacentridae^14,15^, Gadidae^16^, Serranidae^9,17–19^ and Sciaenidae^20–22^. Sound has been shown to play an important role in such aggregations as a species-specific recognition signal, orientation mechanism, and stimulant of sexual activity and synchronous reproductive efforts of the entire colony/aggregation^23,24^. When numerous fish collectively produce sound they can raise the ambient noise levels significantly, producing what has been termed a chorus.

A fish chorus is defined as the continuous sound produced by vocalising fish that significantly raises the background noise level in a characteristic frequency band by >3 dB for an extended period (≈1 h or more) (Cato^25^).

Australian coral reefs have been shown to emanate a wealth of biological sounds, in particular, fish choruses^25–31^. Around Lizard Island in the Great Barrier Reef (GBR), six continuous fish choruses were recently identified across six field sites^31^. Emitting acoustic energy between 50 and 2000 Hz, several of these choruses exhibited distinct temporal patterns, where strong diurnal activity was present in five of the six choruses^31^. Four of the six choruses were predominantly detected at distinct times during hours of darkness, while two were also found during the day. Field sites in the north and south of the island displayed consistently higher chorus diversity and levels than other sites, suggesting that particular locations are important aggregation areas for soniferous fish^32^. Three of these choruses were previously undocumented and could hold valuable information on the source presence, abundance and dispersal patterns.

The aim of this study was to determine the periodicities of selected fish choruses and investigate likely environmental drivers around Lizard Island in order to:

- Elaborate potential areas of essential fish habitat,
- Better understand existing spatio-temporal patterns, and
- Provide a means of comparison with future monitoring, particularly post environmental change or increases in anthropogenic pressure.

## Results

### Spatial patterns of choruses

**Figure 1:**
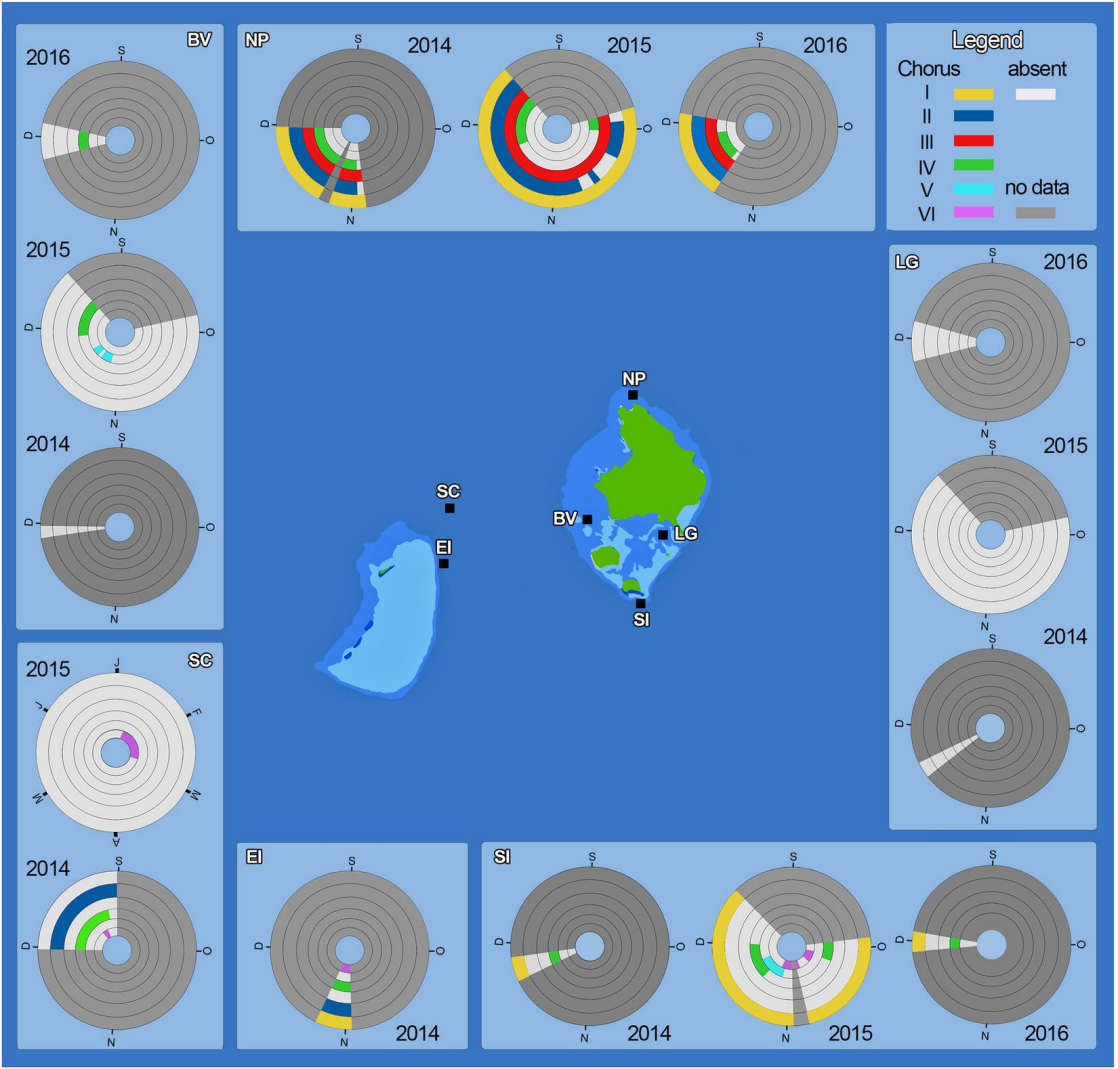
Circular Gantt diagrams of fish choruses identified around Lizard Island. Gantt chart units - calendar days with letters representing months of the year. S to D (September to December), J to J (January to July). Fish choruses are colour coded from outer to inner circles: Chorus I to VI (orange to purple), no chorus (white), no data (dark grey). Map diagrams were designed and created using Microsoft Excel (version 2010) and Adobe Photoshop (version CS6).

**Figure 2:**
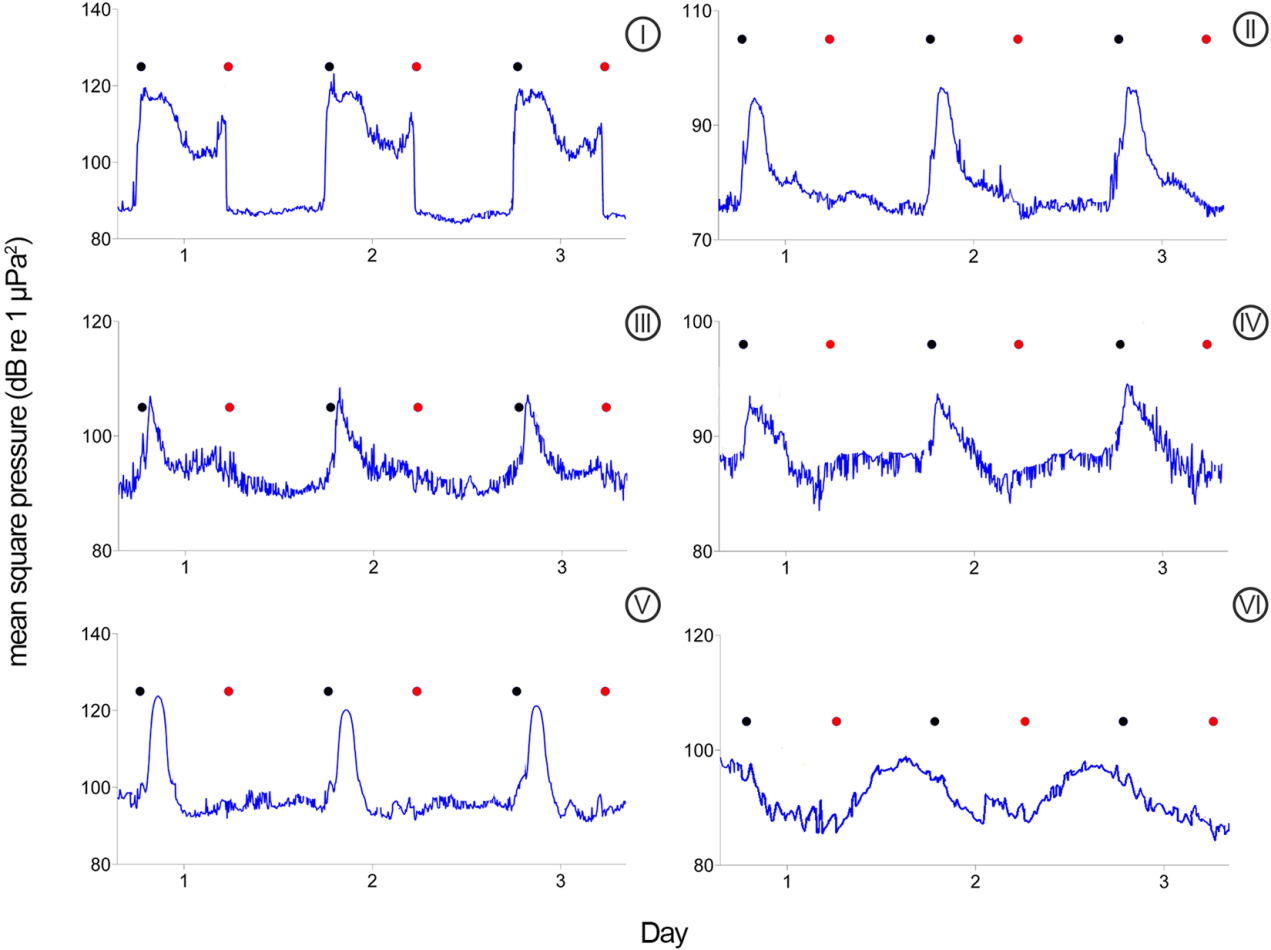
Example of daily periodicities over three example days of fish choruses (I – VI) around Lizard Island. Mean square pressure for Chorus I to VI is in frequency bands 400–700, 340–360, 20–280, 90–150, 150–500, 260–350 Hz, respectively. Black circle = sunset; red circle = sunrise.

From 2014 to 2016, the detected fish choruses exhibited distinct and regular spatial patterns at particular sites around Lizard Island (Fig. 1). The sites of North Point (NP), Eagle Island (EI) and South Island (SI) displayed the highest diversity of fish choruses (a total of four). Chorus I was observed at three sites, NP, EI and SI, where the NP site consistently displayed the highest chorus levels (Fig. 2). This chorus was not detected at the remaining field sites, Big Vicky’s (BV), Lagoon (LG) and Shipping Channel (SC) during any of the recording periods. Chorus II was recorded at three locations, NP, SC and EI, with highest recorded levels at the SC site. Throughout the entire monitoring period, Chorus III appeared only at NP, potentially exclusively active in this area of Lizard Island (Fig. 1). Chorus IV was the most spatially widespread chorus and was only absent from the LG field site. Chorus V was detected at two sites, BV and SI. Chorus VI was observed at three sites, SC, EI and SI (Fig. 1).

These six choruses were some of the most notable bioacoustic contributors to the Lizard Island soundscape. In addition to these, a large number of discrete calls, suspected to be produced by fish, were also consistently observed during analysis^31^. While most of these calls did not occur at a sufficient rate to produce a chorus, two additional choruses (named Choruses VII and VIII), not reported in a previous study^31^ have been detected and included in this study. These choruses were detected at the BV and LG sites. Opportunistic sound recordings revealed that Choruses I and II were also present on the east side of the island.

### Temporal variation

The choruses displayed a diverse assortment of patterns across several temporal ranges, including diel, tidal, lunar and annual time scales.

Inspection of chorus levels of Choruses I to VI revealed notable diel cycles (Fig. 2). A considerable increase in chorus levels post-sunset was observed in all cases with the exception of Chorus VI, which was predominantly present during hours of sunlight. A low signal-to-noise ratio in some of the fish choruses meant that the daily duration of each chorus could not always be measured, particularly in the case of Choruses IV and VI.

Chorus I levels spiked significantly (>30 dB above background levels) around sunset, dropped to ≈100–105 dB re 1µPa for ≈6 h, then spiked again just prior to sunrise (Fig. 2). Daily chorus duration remained largely consistent (≈11–12 h) across the different sites (Table 1) and there were no instances where Chorus I was observed during the day. Chorus II began abruptly around sunset (±20 mins), building to a sharp peak (up to 20 dB above background levels) before dropping below discernible levels after ≈2 h at NP and EI and ≈3 h at SC (Table 1). Chorus III was observed exclusively at NP and commenced around 1 h post-sunset with a duration of ≈1 h. Chorus IV began at sunset, reaching maximum levels 1 h later, before calling activity gradually diminished and later dropped to background levels roughly 2 h after sunrise (Fig. 2). Chorus V, observed at BV and SI for a three-week period in November, 2015, always commenced after sunset, ramping up (>25 dB) to a peak over a ≈90-min period. With each consecutive day during this 21-day period, the start of Chorus V commenced later each evening, with the difference in start time shifting by 120 mins (from ≈19:30 to 21:30) over the three-week period in which it was detected. Chorus VI began ≈1–2 h before sunrise and dropped below observable levels in the early morning, though accurate times were difficult to discern due to low SNR.

**Table 1:** Mean chorus durations (h) with standard deviations (SD) and the number of nights (n) observed. Chorus IV and VI durations are absent due to their low signal-to-noise ratio.

	NP			SI			EI		
I	Mean	SD	n	Mean	SD	n	Mean	SD	n
2014	11.89	0.63	29	11.32	0.34	5	10.99	0.18	8
2015	11.75	0.39	80	11.26	0.47	72			
2016	11.83	0.34	21	10.86	0.32	4			
2014–16	11.79	0.45	130	11.24	0.47	81			
II	NP			SC			EI		
2014	1.79	0.70	29	2.90	0.83	29	1.86	0.42	8
2015	1.82	0.62	47						
2016	2.01	0.47	21						
2014–16	1.85	0.62	97						
III	NP								
2014	1.64	1.08	29						
2015	1.49	0.56	76						
2016	1.71	0.34	16						
2014–16	1.56	0.70	121						
V	BV			SI					
2015	1.82	0.39	14	2.09	0.51	16			

### Tidal and lunar

**Figure 3:**
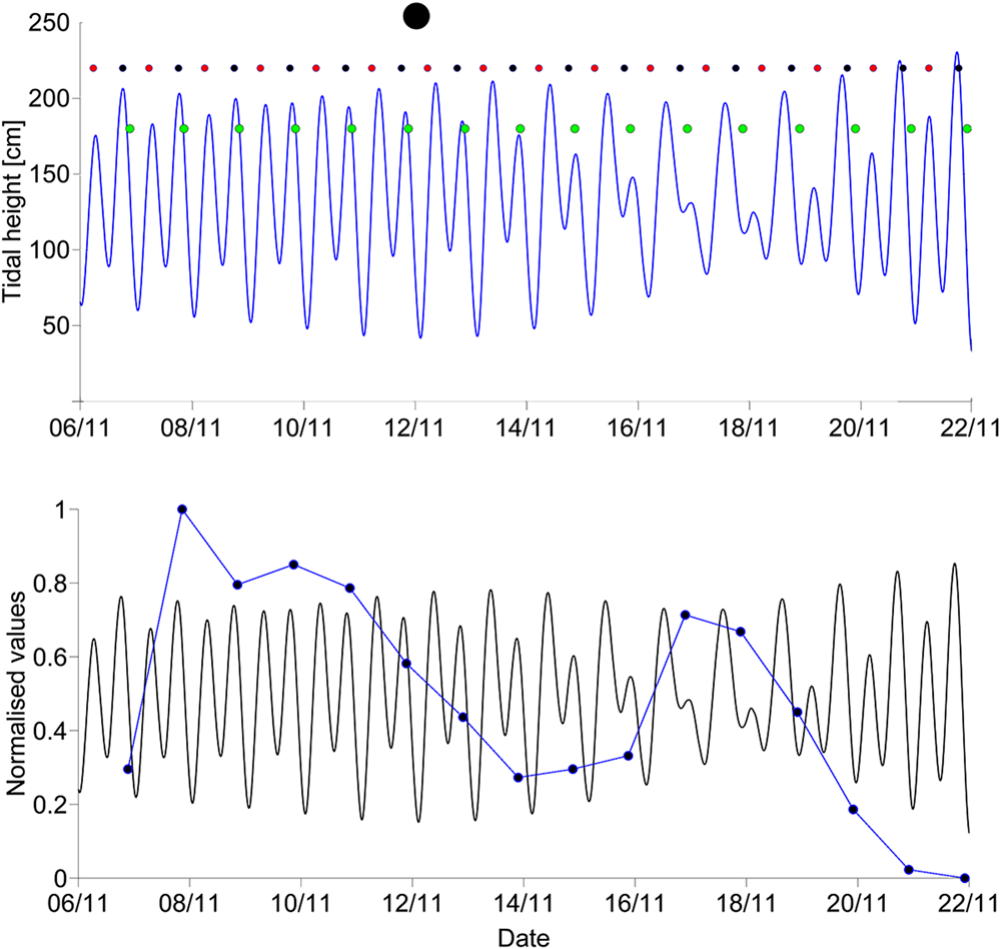
Time of the peak Chorus V levels (green dots) in relation to tide height at SI (A) over a 16-day period in 2015, with sunrise and sunset marked by red and black dots, respectively. Normalised (0–1) peak Chorus V levels (black dots connected by blue lines) with normalised tidal heights (0–1) at SI (B). New moon (large black dot).

Evidence of tidal periodicities was only observed in two choruses. Daily Chorus V peak levels predominantly occurred after a local tide maximum (Fig. 3), 81% of the time (n = 16) at SI and BV, where the chorus was present.

**Figure 4:**
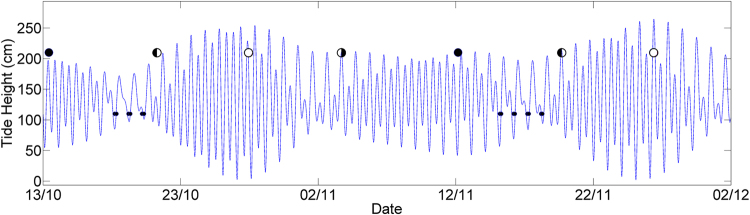
Peak times of previously unidentified fish chorus at the LG site (small black circles) in 2015. New moon (large black circles), first quarter (black/white circles), full moon (white circles) and third quarter (white/black circles).

At the LG site, a discrete and previously unidentified chorus was detected over a short period in October and November. Observed over seven separate nights, the chorus displayed tidal and lunar influences, coinciding with tidal periods and appearing to reach maximum levels around neap tides, or seven days after spring tides (Fig. 4).

**Figure 5:**
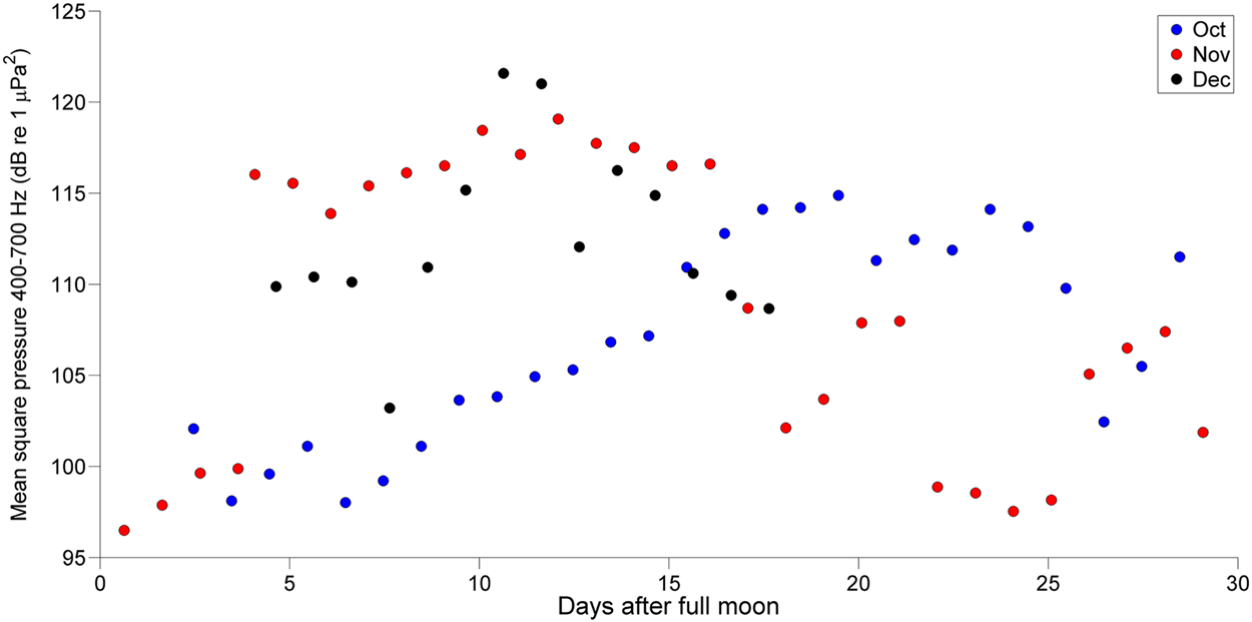
Scatter plot of Chorus I peak levels in relation to lunar period. Mean square pressure (MSP) averaged over 300-s windows.

Similar to the chorus in Fig. 4, lunar periodicities were evident in several of the choruses (I, II and III), with levels increasing around the new moon and decreasing around the full moon. Chorus I, for example, exhibited a negative correlation between days after the new moon and chorus levels in October, which switched to a positive correlation in the subsequent months (Fig. 5 and Table 2).

**Table 2:** Correlation coefficients for Chorus I peak levels and days around new and full moon.

Month	n	Full Moon		New Moon	
		R	p value	R	p value
Oct	31	0.72*	<0.001	−0.718	<0.001
Nov	30	−0.26	0.1581	0.60	<0.001
Dec	14	0.14	0.6285	0.46	0.102

*Three outliers removed from analysis.

### Seasonal

Lizard Island has a tropical climate consisting of an austral summer-wet season and a winter-dry season. Seasonal periodicity was seen in Choruses I to VI across all field sites, with a trend of increasing chorus acoustic presence and activity levels from September onwards, into the summer months (November – January), then decreasing towards the end of the summer months. Intermittent individual calls from these choruses could occasionally be heard during the end of the summer-wet season and start of the winter-dry season (March 2014 and April-May 2015) across sites around Lizard Island, but levels did not form a chorus by definition. Long-term recordings from the SC site (December 14 – July 15) also revealed a comparable pattern of chorus activity to the other field sites. Three choruses (II, IV and VI) were present in December before two of them disappeared prior to January. Chorus VI continued to be observed throughout early January to late February, but was not observed after this period, through until July.

### Annual

**Figure 6:**
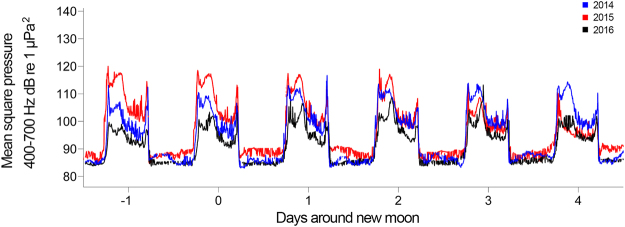
Chorus I levels at the NP site around the November new moon period for each year of recording. Day 1 begins on the first midnight after new moon and time intervals continue on a standard 24 h cycle. Mean square pressure (MSP) averaged over 300-s windows.

Choruses detected in multiple years (I to IV) displayed consistent diel patterns (start/end times), levels (mean square pressure), and comparable durations (Table 1, Fig. 6). Table 1 (mean and SD) shows that chorus durations remained highly consistent over long time scales (2014–2016).

### Chorus frequency bands

The frequency bands that choruses occupied did not deviate over the recording period with the exception of those of Chorus II at NP, which shifted in the final recording year. In 2016, this chorus shifted frequency bands to ≈360–380 Hz, up from ≈340–360 Hz, observed in 2014 and 2015. All other choruses continued to occupy the same frequency bands.

### Environmental variables

The correlation between chorus descriptors and environmental variables displayed evidence of chorus and site-specific relationships (Table 3).

**Table 3:** Correlation coefficients of potential environmental drivers and fish choruses.

site	n	Pk chorus time vs level	Pk chorus level vs tide height	Pk chorus time vs pk tide time	Pk level vs chorus duration	Pk level vs temperature	Pk level vs wind speed	Pk level vs sunlight	Pk level vs moonlight
NP CI 2014	29	0.74***	0.13	0.18	−0.07	0.74***	0.26	−0.49***	−0.17
NP CI 2015	80	0.29**	0.28	0.52***	0.06	0.14	−0.30**	0.28	−0.22*
NP CI 2016	21	0.76***	0.08	0.49*	0.23	−0.39	−0.24	0.3	−0.54**
SI CI 2015	72	0.53	0.51	0.53**	−0.37	0.54	−0.47	†	0.04
NP CII 2014	29	0.77***	0.24	0.33	0.82***	0.32	−0.42*	−0.31	−0.70***
NP CII 2015	80	0.42	0.26	0.42	0.60***	0.32**	−0.35**	†	−0.45***
NP CII 2016	21	0.77	−0.09	0.21	0.37	−0.47	0.09	0.36	−0.72***
SC CII 2014	29	−0.02	0.3	−0.18	0.59***	−0.14	0.14	−0.11	−0.34
NP CIII 2014	29	0.63	0.18	0.3	0.02	0.51**	0.16	−0.41	−0.48*
NP CIII 2015	76	−0.02	0.01	0.38***	0.09	0.02	0	†	−0.55***
NP CIII 2016	16	0.93***	0.57	0.51	−0.68	0.64	−0.71	0.29	0.41
NP CIV 2015	19	0.82***	0.16	−0.01		0.72***	0.43	†	−0.28
SC CIV 2014	26	−0.19	−0.13	0.1	0.65	0.21	−0.17	−0.49	−0.40*
BV CIV 2015	17	0.77***	0.47	0.51		0.85***	0.17	†	0.09
BV V 2015	14	−0.31	−0.41	0.37	0.91***	0.1	−0.27	−0.27	−0.18
SI V 2015	16	−0.65*	−0.13	0.15	0.84***	−0.19	−0.57*	−0.18	−0.67***

***p < 0.001, **p < 0.01, *p < 0.05. ^†^Limited data (missing env. variable measurements).

At individual sites, several of the choruses displayed a positive relationship with water temperature. Chorus I peak levels for 2014 at NP were significantly correlated with water temperature (r = 0.74, p < 0.001). In 2015, Chorus II and IV peak levels at NP and BV were also positively correlated with temperature (Table 3). Temperature was positively correlated with Chorus III in 2014, 2015 and 2016, but in the second recording year the positive correlation was not significant. All choruses (peak SPL’s) were, to varying degrees, negatively correlated with moonlight levels with three exceptions (Chorus I at SI in 2015, Chorus III at NP in 2015 and Chorus IV, BV in 2015) [Table 3]. Chorus II displayed the strongest (negative) correlations with moonlight (2014–2016), followed by Chorus V at SI in 2015 (Table 3). Chorus I was negatively correlated with moonlight levels at NP throughout recording years, but showed a much weaker positive correlation at SI in 2015 (r = 0.04). Wind speed and peak chorus levels showed a negative relationship in 9 out of 16 chorus site measurements (stronger winds equalling lower chorus levels), where the correlation was strongest at NP for Choruses I-III and Chorus V at SI (Table 3). Sunlight levels, taken as daily averages, were positively and negatively correlated with peak levels for Choruses I-V. However, correlation was only statistically significant for Chorus I at NP in 2014. Time of chorus peak relative to sunset was significantly correlated with peak chorus level in several choruses, particularly Chorus I from 2014 to 2016, illustrating that peak chorus levels occurred consistently at a similar time of the evening (Table 3). However, Chorus V peak levels did not occur at a similar time each night but shifted to later times in the evening, peaking on a falling tide and as a result, there was limited correlation between the time of peak chorus and the chorus levels alone.

A weak positive correlation between peak chorus levels and tide height was seen in four of the six choruses but none were found to be significant. Peak tide time and chorus level showed a stronger relationship, with significant correlations for Choruses I and III, but not for Chorus V. Peak chorus level and chorus duration were significantly correlated in two of the six choruses, Choruses II and V. The chorus peak mean square pressure levels were higher when the chorus continued for a longer period of time.

## Discussion

The fish choruses detected in long-term recordings around Lizard Island have shown various levels of spatial delineation and distinct temporal patterns. Three of these choruses have previously been speculated to originate from bigeye fish *Pempheris adspersa* ^33^ (or other Pempheridae, Chorus I), Batfish^30^ (*Platax sp*., Chorus IV) or members from the Sciaenidae, Scorpaenidae, Callionymidae (stinkfishes) and Aridae^26^ (catfishes) (Chorus VI), while the sources of Choruses II, III and V currently appear undocumented^31^. The delineation of spatio-temporal patterns for such a variety of species, in such a small area, emphasises the importance of documenting their characteristics and occurrence, particularly in a World Heritage area, that is subjected to environmental pressures^34^.

The consistency and regularity of these choruses suggests high site fidelity around Lizard Island, with several sites simultaneously hosting multiple choruses, providing evidence that particular areas have significant biological importance for vocalising fish. A number of studies have shown that reef fish can maintain long-term site fidelity, with some spawning sites exhibiting multi-decadal use^35,36^ and a recent global baseline of fish spawning aggregations found that up to approximately 90% of aggregation records were from reef pass channels, promontories and outer reef-slope drop-offs^4^. Several aggregation sites of spawning coral trout (*Plectropomus leopardus)* have been identified around Lizard Island and notably, individuals displayed strong fidelity to their chosen aggregation site^37^. Behaviours like these may in part explain NP’s higher levels of chorus diversity, as the area contains multiple seascape features thought to be favourable to aggregating fish^38^. Anecdotal evidence from the opportunistic, short-term recordings (omitted from the long-term analysis) made on the east side of the island, confirmed the presence of Chorus II, making the absence of Chorus II at SI and BV an interesting observation. Chorus III was only observed at the NP site, in contrast to Chorus IV, which exhibited a widespread presence around the island. These differences in chorus presence imply a single aggregation site for the former and that several distinct aggregations form around the island group in the latter. Chorus V was observed at BV and SI, displaying the highest chorus levels at SI, suggesting that this area in particular, is favoured as an aggregation zone for this species. Chorus VI appeared to be confined to deeper sites, with the exception of NP. Levels were highest at SC suggesting a species that aggregates in open water areas, or in number sufficient to be heard from nearby Eagle Island.

None of the six choruses were observed at the LG site on the east of the island. This is likely to be due to a combination of acoustic and ecological parameters. The site’s shallow water depths can cause acoustic signals to attenuate more quickly than at the deeper sites, therefore reducing the sampling area compared with deeper sites. The site is also virtually isolated from the open sea with the exception of a narrow (75 m) channel entrance. From an ecological perspective, if choruses are associated with fish spawning, the lagoon, with its sheltered, shallow habitats and large numbers of resident predators, would not be considered advantageous for egg survival. Historical spawning aggregation records document several locations around Lizard Island, including SI, but omit LG, providing weight to this hypothesis^37,39^. Through all analysis, the gaps in sampling periods, presented in this study necessitate that conclusions on spatial patterns of choruses should be treated with a degree of caution, until further recordings can be made.

All six choruses exhibited regular diel periodicity across the recording period, and the majority of chorus activity occurred at night. This provides strong evidence that five out of the six choruses are produced by nocturnal fish species. Diel periodicity in fish choruses has been observed in several other locations including the eastern Indian Ocean and Timor Sea^25,26,40^, Southern Atlantic^41^, the central Pacific^42,43^, the Mediterranean^44^, the Arabian Sea^45^ and the mid-Atlantic^46^, several of which begin early evening, increasing at sunset, peaking a few hours after sunset and ending by sunrise^28,32,40,46^.

The weak correlation with tidal patterns, especially for Chorus I, whose daily peak levels did not display any changes in tidal regimes, suggest tide has little influence on the aggregation function. However, while not statistically significant, likely due to the limited data and multiple drivers, Chorus V displayed a possible tidal influence, where peak chorus levels occurred consistently on falling tide at the two sites it was present. Chorus V was present for a short three-week period in November, where peak levels were highest around the spring tides. This may be indicative of a reproductive strategy, where timing of spawning coincides with strong ebbing tides, in order to maximise tidal flushing of eggs to offshore waters^47^. Large numbers of fish have also been observed migrating to outer reef edges and forming spawning aggregations 1.5–4 h after the high tide, showing a similar tide-related pattern seen in Chorus V^39^.

Many coral fish species are thought to exhibit moon-related reproductive patterns because periodic variations of photoperiod and water temperature are less distinct in equatorial and tropical zones compared to temperate zones ^7^. Most of the choruses displayed negative correlation of chorus peak levels with moonlight, indicating that activity is higher around the new moon when lunar light levels are minimal. Change in moonlight intensity has been found to be a stimulating cue for gonadal development and gamete release in fish, where moonlight levels can inhibit activity in some species and promote it in others^48^. Previous studies of fish spawning aggregations around Lizard Island found several instances where these events occurred around the new moon^37,39,47^. Evidence of moon phase influence on fish choruses has also been noted in other areas, including off Darwin, at Scott Reef and around the Maret Islands in north Australia^28,30,40^.

Lunar and semi-lunar periodicity was not a pronounced feature in five out of the six choruses in 2015 when analysing the recording period as a single event. However, when recording periods were split into months, trends became more visible. The change from negative to positive correlation with Chorus I levels and lunar state from October to November and December may indicate the commencement of a peak in spawning activity for this species. Spawning at time with low lunar light levels is a reproductive strategy used by several species to decrease predation risk and enhance egg survival through tidal dispersal^39,49^. In contrast to the other choruses, the previously unidentified chorus at the LG site, active for a total of six days after the full moon, showed a strong lunar pattern. However, the limited presence of many of the choruses, e.g. Chorus V for just over three weeks, means that it was not always possible to elucidate lunar patterns without longer datasets. Therefore, larger sample sizes are required to determine the significance of these patterns, particularly for those which only appear for a few days in a few months. Currently, such datasets are not yet available at Lizard Island.

Over the year, fish choruses displayed strong seasonal periodicity, displaying intermittent presence. Chorus activity at sites around Lizard Island was considerably higher in months of the austral wet season than the earlier months at the end of the dry season. Chorus activity was minimal at all the long-term field sites near the beginning and middle of the austral dry season (March to July). A chorus found in the northern GBR closely resembles Chorus VI in duration, frequency and timings also displayed strong seasonal periodicity, with similar high calling rates over wet season^26^. A recent review of fish that form spawning aggregations found that the spawning season of most reef species typically lasts for less than three months of the year^4^. This seasonal periodicity is thought to have evolved as a reproductive strategy, to ensure optimum spawning conditions and has been observed in several coral reef fishes, particularly in relation to temperature thresholds^6,9^. Temperature has been shown to have a pronounced influence on fish behaviour and physiology, particularly reproduction^9,50,51^. Several species that form spawning aggregations (Serranidae) have strong associations with water temperature, such as coral trout (*Plectropomus leopardus*) which spawn along the GBR once water temperatures reach 26 °C^6^ or the Nassau grouper (*Epinephelus striatus*) in the US Virgin Islands when ambient water temperatures are between 25 and 25.5 °C^9^. Sound production can also be functionally related to ambient water temperature because the majority of fish are ectothermic so that muscle activity, used for generating sound, in some species can be influenced by temperature^46^. Fish chorus activity was also found to be significantly related to temperature in northern Australia^40^.

Inter-annual variations in chorus levels are likely to be related to a combination of environmental conditions. In particular, the relative timing of temperature cycles, tidal periods and lunar phases, which have been shown in this study and previous work to have pronounced effects on vocalising fish^25–28,40,52^ can have a combined effect. Interestingly, most of the fish choruses were present at the same sites each year suggesting that species are either resident reef populations or transients that annually migrate to specific locations around Lizard Island.

Some environmental variables can have a confounding effect on either sound production or detection, or both, predominantly through the generation of geophysical noise or water movement^53^. Wind speed appeared to have a strong negative relationship with peak chorus levels for the majority of choruses. Currents in shallow water areas around Lizard Island are predominantly driven by wind^54^. Wind-driven current fluctuations may influence fish choruses in several ways. High levels of water movement may require insupportable levels of energy consumption to form large fish aggregations and smaller aggregations therefore have lower chorus levels. Strong currents may reduce fertilisation success rates in a spawning aggregation by removing eggs before they are fertilised. However, currents play a key role in dispersing fertilised eggs, demonstrating the importance of timing in spawning events. Estimating current speeds over a large area would require significant logistical effort. Yet, if achieved, this would potentially allow chorus attributes to be used as potential indicators of spawning success. Wind may affect the dispersal patterns of marine fauna like fish larvae and zooplankton, on which soniferous planktivorous fish feed so that calling rates and levels are minimised to conserve energy in the absence of food^55,56^. Rain was omitted from the statistical analysis as an explanatory variable due to limited rainfall at Lizard Island during the main recording periods.

A major aggregation site for coral trout is located on the west side of BV, close to the recording site, where spawning activity was reportedly highest highest during new moon periods in November^37^. This coincides with Chorus V spatial and temporal activity, making it plausible that coral trout are the source of this chorus. While no direct recordings of coral trout vocalising have been reported, several members of the Serranidae family are known sound producers^9,17–19^. The frequency band of Chorus V was approximately 150–500 Hz. The low frequency band could suggest that the chorus was produced by a large vocalising fish as to produce sounds at lower frequencies in fish are often associated with a greater body size^57^.The spectral content of a call, including the spectral peak, is related to a combination of swimbladder size, sonic muscle size and the mechanism by which the muscle impinges on the swimbladder^57,58^. The relationship is such that larger fish, with more developed sonic muscle blocks tend to produce calls of higher source level and lower frequency, than smaller or less developed fish of the same species. However, this relationship becomes more complicated if multiple twitches of the swimbladder are used to produce calls from a train of pulses. Further work is therefore required to establish the source of Chorus V.

This study has revealed a diverse range of information regarding the spatial and temporal patterns of fish choruses around Lizard Island. From a spatial perspective, the high site fidelity displayed by fish choruses contributes to our understanding of population spatial dynamics, potentially revealing aggregation hotspots and dispersal patterns of soniferous fishes, which are not well documented^43^. Fish that aggregate have an increased vulnerability to exploitation, which has been demonstrated in several large aggregating species, notably the Jewfish, *Epinephelus itajara* and the Nassau grouper, *Epinephelus striatus* ^59,60^ and black jewfish, *Protonibea diacanthus* ^61^. In the Caribbean, the Nassau grouper has experienced dramatic population declines from overfishing and has recently been placed on the endangered species list. Determining with passive acoustics where fish aggregations occur can help develop ecosystem models of fish populations, guide seasonal closure of key fish spawning areas, placement of future marine reserves and estimation of fish stocks, thereby ensuring the survival of exploited species^3,20,21,46^.

By identifying temporal patterns in fish choruses and determining their drivers we can greatly improve our understanding of marine soundscapes. Understanding and quantifying periodicities of fish choruses over a wide range of temporal scales from hours through to years improves occurrence, abundance and distribution estimates of reef fish, and collectively, the ecology of their corresponding ecosystems. Quantifying environmental periodicities also helps to improve monitoring methodology and has particular relevance for ecological monitoring and assessments, whose accuracy strongly depends on quantifying these rhythms. This in turn, benefits marine protection strategies by maximising locality and timing of management actions.

The repeating spatio-temporal patterns exhibited by several choruses highlight their potential as long-term monitoring reef indicators. Traditionally scarce due to the challenges of surveying highly complex systems for prolonged periods, long-term indicators of reef health are required following two successive years of widespread coral bleaching, which has caused mass coral mortality throughout the GBR^34,62^. However, the relatively short-term spatio-temporal patterns in choruses that were detected around the islands demonstrate that a single recording site alone cannot adequately be used to represent a complex reef soundscape and that long-term recordings across a range of sites are key to accounting for variations that can occur in the soundscape. This reinforces the necessity for establishing multiple on-going field sites. While these variations are significant, even in the short-term, coral reef soundscapes are highly complex acoustic environments, where hundreds of sound sources, many of biological origin, can be recorded over a short period from a single location. Identification of individual signals contributes significant knowledge to understanding species diversity within the soundscape, yet classification of sound sources is a challenging and time consuming task. Automated signal recognition and source identification show great promise, yet remain in developmental stages^63–65^.

## Conclusion

The coral reefs surrounding Lizard Island in the GBR have a diverse soundscape that contains a plethora of bioacoustic phenomena, notably biological choruses produced by fish. Six identified fish choruses exhibited spatial and temporal patterns around Lizard Island from 2014 to 2016. Several choruses displayed high site fidelity, indicating that certain sites may represent important habitat for fish species, such as fish spawning aggregations sites.

The choruses displayed a broad range of periodicities, from diel to annual. This information provides new insights into the ecology of vocalising fish species and the surrounding reef ecosystem. All choruses were affected by one or more environmental variables including temperature and moonlight, the latter of which had a significant influence on the timing and received levels.

Results from this work highlight the application of passive acoustic monitoring (PAM) for long-term monitoring of coral reefs. This is highly relevant following recent extensive global disturbance events, particularly coral bleaching.

## Methodology

### Study area

**Figure 7:**
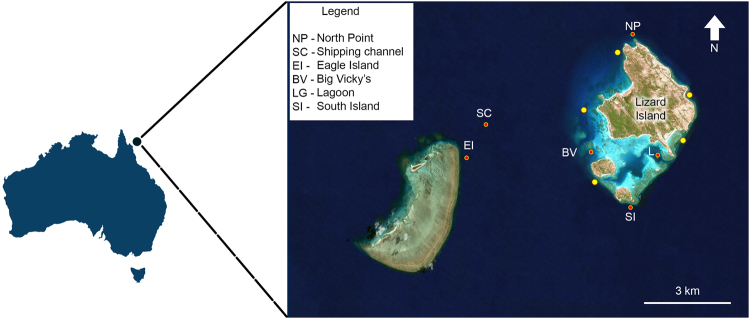
Location of field sites around Lizard Island. Red dots denote long-term sites and yellow dots denote location of opportunistic recordings. Long-term site depths (m): NP [18–21], BV [7–10], LG [6–9], SI [13–16], SC [25–30], EI [19–22]. Refer McWilliam^31^ for more information. Map image: Google Earth imagery, © 2017 DigitalGlobe Image Landsat/Copernicus.

Lizard Island (14°40.88′S, 145°27.82′E) is a continental island located 270 km north of Cairns and approximately 30 km off the coast, within the World Heritage listed GBR. There are three other smaller islands: Palfrey, South Island and Bird Island nearby, which make up the Lizard Island Group (Fig. 7). Coral reef borders the smaller islands and a narrow band of fringing reef surrounds much of Lizard Island. Between Lizard and South Island, a more extensive reef encompasses the Blue Lagoon, with water depths to 12 m. The maximum tidal range at Lizard is ± 3 m. Current speeds vary around the island group with the entrances to the lagoon and waters around North Point experiencing current speeds that can exceed 30 cms^−1^ during tidal cycles^54^.

The island is subjected to south-easterly trade winds from April to September with maximum monthly wind speeds averaging 42 km/h for this period^54^. In the summer months, wind speeds drop considerably, averaging 28 km/h and become more variable in direction. Gusty north-westerly winds are interposed with calm periods in November and December.

### Data collection

A series of underwater audio recordings were obtained at various times over a three-year period from six field sites around Lizard Island (Fig. 7 and Table 4). Recordings were made using a) SoundTrap 202 (Ocean Instruments, New Zealand) digital sound recorders with a 48 ksps sample rate (manufacturer’s specifications of a flat response within ±3 dB between 20 Hz and 60 kHz) and b) an underwater sound recorder (USR) (developed by the Centre for Marine Science and Technology (CMST) at Curtin University and the Defence Science and Technology Organisation^66^) with a calibrated omni-directional, HTI 96-min hydrophone (HighTech Inc., MS, USA) with a 18 ksps sample rate.

**Table 4:** Details of acoustic recordings, continuous (C) or duty cycle (%) taken around Lizard Island from 2014 to 2016.

Year	Site	Equipment	Sampling type	Dates	Total number of recording days
2014	North Point	ST	C*	29/10–08/11	10
		ST	16.67%	10/11–17/11	7
		ST	C	18/11–30/11	12
	Eagle Island	ST	C	31/10–8/11	8
	South Island	ST	C	22/11–27/11	5
	Big Vickys	ST	C	28/11–30/11	2
	Lagoon	ST	C	18/11–21/11	3
	Turtle Beach	ST	C	02/11–06/11	4
	South Bay	ST	C	21/11–24/11	3
	Crystal Cove	ST	C	06/11–08/11	2
	Coconut Beach	ST	C	29/10–01/11	3
	Palfrey	ST	C	19/11–22/11	3
	Shipping Channel	USR	20%	01/12–31/12	31
2015	North Point	ST	C	07/03–16/03	9
		ST	C	26/09–15/12	80
	South Island	ST	C	30/09–15/12	76
	Big Vickys	ST	C	06/03–17/03	11
		ST	C	27/09–16/12	80
	Lagoon	ST	C	06/03–15/03	9
		ST	C	26/09–13/12	78
	Shipping Channel	USR	20%	01/01–05/07	186
2016	North Point	ST	C	07/04–07/05	30
		ST	93%	12/11–27/11	15
		ST	C	20/11–28/11	8
		ST	C	27/11–03/12	6
	South Island	ST	C	07/04–23/04	16
		ST	C	29/11–03/12	4
	Big Vickys	ST	C	07/04–07/05	30
		ST	C	26/11–04/12	8
	Lagoon	ST	C	07/04–07/05	30
		ST		26/11–04/12	8
	Palfrey	ST	C	28/11–04/12	6
				Grand total	783

*ST: Soundtrap (48 ksps sample rate). **USR: Underwater Sound Recorder (18 ksps sample rate).

Each SoundTrap was piston-phone calibrated with a known level of 121 dB re 1µPa at 250 Hz by the manufacturer, while the USR was calibrated across its full frequency spetcra with a white noise generator at −90 dB re 1 V^2^/Hz. For each deployment, a Garmin 60Csx GPS unit and a laptop with internet connection were used to ensure continuing SoundTrap clock accuracy and to minimise clock-drift readings. Field recording sites were selected to be representative of the variety of seascapes found around Lizard Island. Site locations were recorded with a Garmin 60Csx GPS, accurate to ±3 m.

SoundTraps were fastened to weighted mounts and diver-deployed to the seabed on sandy substrates, at a minimum distance of 3 m from reef structures. Recorders were collected and redeployed every 10–14 days for data acquisition and battery recharge. A second SoundTrap was deployed for a period of around 5 mins prior to retrieval of the first SoundTrap, to provide adequate cross-over time. Spectrograms of the overlapping *. *wav* files were visually and audibly inspected to determine an appropriate cross-over point in SoundTrap wav files, i.e. where diver and handling noise were lowest (minimal). To aid equipment relocation in turbid conditions, a sub-surface marker composed of two concrete breeze blocks and a rope attached to a sub-surface buoy were placed ≈5 m from each SoundTrap.

The USR was deployed off the side of one of Lizard Island’s research vessels (Kirsty K). The recorder was gently lowered to the seafloor by a rope pulley system, followed by a 100 m rope line with a 60 kg sacrificial dump weight in order to maximise the distance of the hydrophone from potential sources of extraneous noise. An acoustic release with sub-surface buoys was attached to the dump-weights for surface-based retrieval of the USR.

In addition to the long-term acoustic data, acoustic recordings were collected opportunistically, at a number of locations around the islands for increased spatial coverage of soundscapes. Recordings ranged from approximately 1 to 3 days in duration (Table 4).

### Audio analysis

Acoustic datasets were analysed using a combination of long-term spectrogram visual analysis and audio inspection of recordings. Calibrated power spectral density (PSD) averages were computed over 300-s windows and joined chronologically to create long-term spectrograms with time on the x-axis and frequency on the y-axis (logarithmic or linear scale), with colour representing power. A Graphical User Interface (GUI) toolbox, CHaracterisation Of Recorded Underwater Sound (CHORUS^67^) was used to display and inspect the spectrograms in the MATLAB software environment (The Mathworks Inc. Boston, MA). CHORUS was designed to analyse long-term underwater sound recordings. The GUI allows the spectrogram adjustment from a single day to several months. Local sunrise and sunset times (when the upper edge of the sun’s disk touches the horizon) were determined with a custom MATLAB routine, which utilises an algorithm developed from expressions by Dogget^68^.

Previous work^31^ identified signals contributing to six different choruses as originating from fish, based on their similarity in frequency content, energy level, duration and temporal patterns of other reported calls and choruses^26,30^, on occasion with sufficient similarity to infer the source species, or family. This study elaborates on the spatio-temporal patterns exhibited by those six choruses utilising the measured duration (start and end time established as the times when ambient energy levels were raised by 3 dB and then maintained for a minimum of 1 hr in the designated chorus bandwidth, e.g. 400–700 Hz), spectral content and frequency of spectral peak of each chorus. Each of these descriptors were measured directly from the long-term spectrograms in CHORUS. The pulse repetition rate of individual calls (where identifiable) and evidence of frequency partitioning between two choruses were determined by visual analysis of spectrograms or waveforms and contributed to categorising each fish chorus. This study includes two additional chorus types not identified in an earlier publication^31^, which were also distinguished using these methods.

### Environmental variables

The time difference (ΔT) between the chorus descriptor and environmental factors was determined for the following combinations:

- Peak chorus time vs peak level*.
- Peak chorus level vs tide height.
- Peak level vs chorus duration.
- Peak chorus time vs peak tide time.
- Peak level vs water temperature.
- Peak level vs wind speed.
- Peak level vs sunlight.
- Peak level vs moonlight.
- Peak tide time vs time of sunset.

*Peak chorus level refers to the highest received level across the selected frequency bands (e.g. 340–360 Hz), i.e. peak band level.

Hourly tidal prediction measurements were taken from the 2016 Maritime Safety Queensland Blue Book^69^ and imported into MATLAB. Five-minute tidal predictions were estimated using the interp1.m function, employing a cubic spline order to incorporate the sinusoidal pattern of tidal levels^70^. Wind speed, water temperature, sunlight and rainfall readings were collected from the Facility for Automated Intelligent Monitoring of Marine Systems’ (FAIMMS) Sensor network run by the Australian ‘Integrated Marine Observing System’ (IMOS). Temperature readings were measured using a Sensus Ultra temperature sensor (ReefNet Inc.) in 10.1 m depth (14°41′17.41″S, 145°26′33.00″E). Datasets and instrument information are available from the IMOS online data portal (www.imos.gov.au). In 2015, HOBO Pendant® Temperature/Light data loggers were deployed at each field site while the FAIMMS network was temporarily offline. Wind speed and direction measurements were obtained from the IMOS Lizard Island weather station. In 2015 and 2016, two additional autonomous anemometers (LEWL-PRO) were deployed north and south of Lizard Island to compare localised wind speeds around field sites. Anemometers were programmed to take a wind speed and direction measurement every five mins. Moonlight levels were estimated using an astronomy and astrophysics package for MATLAB^57,71^. Lunar periods in relation to chorus peak levels were collected from online open access datasets (https://www.timeanddate.com) and processed in MATLAB. Peak chorus levels and tidal heights were normalised using a min-max normaliser for graphical presentation purposes. This linearly rescales every feature to the (0, 1) interval using equation 1, where *z* represents tidal height (cm) or peak chorus level (dB):

1 $$z=\frac{{\rm{x}}-\,{\rm{\min }}({\rm{x}})}{[\,\max ({\rm{x}})-\,{\rm{\min }}({\rm{x}})]}$$

### Temporal comparison

To account for the strong lunar influence on fish chorus levels, inter-annual comparison of fish choruses was carried out by aligning time series to the day of either the previous full or new moon^54^. Short-term comparisons could then be conducted by comparing consecutive lunar periods and diel patterns were investigated by zero-ing data to sunrise, sunset or tidal peaks. Correlation coefficients were computed in MATLAB to investigate potential relationships between fish choruses and environmental variables.

### Data availability

The datasets generated during the current study are available from the corresponding author on reasonable request.
